# Patterns of Drug Use Across Social and Sexual Contexts in Spain: Insights From the European Web Survey on Drugs 2024

**DOI:** 10.1007/s10508-026-03525-y

**Published:** 2026-09-27

**Authors:** Lucia Gallego-Deike, Marcos Bella-Fernández, Luisa María López-Sánchez, Begoña Brime-Beteta, Silvana Ruiz-Medina, Hilario Blasco-Fontecilla

**Affiliations:** 1 Instituto de Investigación, Transferencia e Innovación (ITEI), Universidad Internacional de La Rioja, Calle José Abascal 51, 28010 Madrid, Logroño, España Spain; 2 Instituto de Salud Mental y Bienestar Emooti, Madrid, Logroño, España Spain; 3 Faculty of Health, Universidad Internacional de La Empresa, Madrid, Spain; 4 Faculty of Psychology, Universidad Pontificia de Comillas (UPC), Madrid, Spain; 5 Observatorio Español de las Drogas y las Adicciones, Delegación del Gobierno para el Plan Nacional Sobre Drogas, Ministerio de Sanidad, Madrid, Spain; 6 Center of Biomedical Network Research On Mental Health, Madrid, Logroño, España Spain

**Keywords:** Drug use, Sexualized drug use, European Web Survey on Drugs, Public health, Chemsex

## Abstract

Drug use is a global public health issue. Sexualized drug use (SDU) involves intentionally using substances to enhance sexual activity across diverse contexts, but it lacks a unified definition. Chemsex is a specific SDU subset that features stimulant use (methamphetamine, GHB/GBL, mephedrone) in prolonged sex group sessions, predominantly among men who have sex with men (MSM). We analyzed data from 6,399 valid participants residing in Spain who completed the European Web Survey on Drugs. Our focus was on sex-related drug use, including chemsex. Recreational use was the most common reason for drug consumption (78.01%). Cannabis and heroin were frequently used to relax or manage stress (49.73% and 30.14%, respectively), improve sleep (26.66%), and cope with anxiety and/or depression (26.35%). Drug use to enhance sexual performance and pleasure was reported by 13.66% of participants, while chemsex use was lower at 3.31%. Though historically associated with MSM, chemsex use is increasingly reported among women. Methamphetamine, cannabis, and MDMA were more frequently used by men to enhance sexual experiences, with methamphetamine and new psychoactive substances being primarily linked to chemsex parties. Illicit drug use in Spain is predominantly recreational and typically occurs at home, festivals, or nightlife venues. Chemsex, particularly among MSM, is an emerging issue associated with sexual, physical, and mental health risks. Addressing this requires targeted prevention strategies, including health education, harm reduction, inclusive care services, and professional training to reduce associated harms and improve health outcomes.

## Introduction

Drug use is a pervasive public health issue worldwide. In Spain, illegal drug use appears to be stabilizing, despite a recent downward trend observed among younger age groups (Ministerio de Sanidad, 2024). Cannabis and cocaine are the most commonly used illicit drugs. Around 12.5% of the Spanish population between 15 and 64 years consumed illicit drugs during the last 30 days (Observatorio Español de las Drogas y las Adicciones, 2024). The use of illicit drugs supposes a cost of roughly 1.5 billion euros per year (Rivera et al., 2017), and in 2023 it took the life of 1360 people in Spain (Observatorio Español de las Drogas y las Adicciones, 2024). Among the various patterns of drug use, it is important to stress that sexualized drug use (SDU) and chemsex are matters of concern for public agencies and are attracting a growing interest in both academic and public health fields.

SDU, also referred to as drug use in a sexual context, encompasses various forms of substance use practices associated with sexual activity, including drug use among female sex workers and their clients, individuals who consume drugs while watching pornography, those involved in swinger environments, and chemsex. In each of these contexts, differences can be observed in the substances of choice, routes of use, acquisition channels, duration of the activity, number of sexual partners involved, prevalence of sexually transmitted infections and associated transmission risk, among others (Curto et al., 2020). However, there is no consensus definition of SDU. It is generally conceptualised as an umbrella term referring to the intentional use of drugs to enhance or facilitate sexual activity, practiced by individuals of any gender identity or sexual orientation, although disproportionately represented among sexual-orientation and gender-minority groups (Muschialli et al., 2025; Śniadach et al., 2025). This lack of consensus may limit the scope of research, which often focuses on Western settings and views SDU primarily through a public health perspective, emphasizing risks to sexual health while potentially overlooking broader demographic, geographical, and cultural diversity (Muschialli et al., 2025).

The term chemsex originated in the United Kingdom and combines the words chems and sex, referring to drug use within a specific sexual context. Initially, the term was used to describe a form of recreational drug use closely associated with gay sexual culture. Although the seminal work by McCall et al. (2015) did not frame this as a defining criterion, the prevailing consensus has been that chemsex predominantly involves men who have sex with men (MSM). Consequently, the majority of research has focused on chemsex within MSM populations (Curto et al., 2020; Maxwell et al., 2019; Wang et al., 2023). Despite this focus, no single, universally accepted definition of chemsex exists, and its scope varies across studies. It is commonly understood as the intentional use of specific psychoactive substances before or during sexual activity to intensify or prolong sexual experiences, often facilitated by geosocial networking applications or occurring in private residences or sex-on-premise venues (Muschialli et al., 2025; Śniadach et al., 2025). While originally documented primarily among MSM, emerging evidence indicates that chemsex also occurs among heterosexual men and women within party and club subcultures, although research on these groups remains limited (Śniadach et al., 2025). Notably, chemsex is not currently recognized as a distinct diagnostic entity in classification systems such as the ICD-11 (Śniadach et al., 2025).

Drugs types used in chemsex include mephedrone (Bourne et al., 2014; Dolenguevich-Segal et al., 2017; McCall et al., 2015), GHB/GBL (Dolenguevich-Segal et al., 2017; McCall et al., 2015; Śniadach et al., 2025), methamphetamine (Dolenguevich-Segal et al., 2017; McCall et al., 2015; Śniadach et al., 2025), MDMA (Dolenguevich-Segal et al., 2017; Śniadach et al., 2025), cocaine (Dolenguevich-Segal et al., 2017; Śniadach et al., 2025), and ketamine (Dolenguevich-Segal et al., 2017; Śniadach et al., 2025). Additional substances may include alkyl nitrites (poppers), PDE-5 inhibitors (e.g., sildenafil), 1,4-butanediol, fentanyl and other opioids, kratom, sodium nitroprusside, and α-PVP. Polydrug use is common and varies across regions depending on availability, cultural context, and local drug markets (Śniadach et al., 2025). While chemsex may occur in one-to-one encounters, it is also frequently practiced in organized or improvised group settings, often referred to as chemsex parties, which have become a recognized element of chemsex culture (Santoro et al., 2020).

Chemsex carries significant risks to mental and physical health. It is linked to unsafe sexual practices, increasing HIV and hepatitis C transmission (Curtis et al., 2019; González-Baeza et al., 2018; González-Recio et al., 2024). Also, some drug use methods, such as slamsex, are themselves high-risk behaviors (Dolenguevich-Segal et al., 2019; Knoops et al., 2022). Non-consensual sexual encounters, driven by drug-induced disinhibition, can cause psychological trauma (Drückler et al., 2021; Ratner et al., 2003). Sustained chemsex users often face depression, anxiety, sleep disorders, psychotic symptoms, and cognitive decline (Batchelder et al., 2017; Bohn et al., 2020; O’Cleirigh et al., 2015).

Slamsex is the intravenous use of drugs, mainly methamphetamine or new psychoactive substances (NPS), within chemsex contexts to intensify sexual experiences. This practice produces a rapid and potent “high” via direct bloodstream injection, a feature that may contribute to its continued prevalence in particular subcultural contexts (Knoops et al., 2022; Ministry of Health, 2024). Although both chemsex and slamsex carry serious physical and psychological risks, slamsex is more damaging due to its intravenous route, which increases the likelihood of addiction, psychosis, neurological harm, overdose, and sexually transmitted infections. Low harm perception and limited mental health support amplify these issues, making chemsex and slamsex a public health concern in Spain (González-Baeza et al., 2018).

This article aims to explore SDU, including chemsex-related practices, in 6399 respondents of the most recent data from the European Web Survey on Drugs (EWSD) residing in Spain who reported illicit drug use. The EWSD is a survey conducted every three years by the European Union Drugs Agency, as part of its effort to gain a deeper understanding of drug use patterns in Spain, with the ultimate objective of reducing drug consumption. Spain has participated in the EWSD since 2021. The survey is directed to residents in the European Union above 18 years old who have consumed illegal drugs (European Union Drugs Agency, 2024). It includes questions on the frequency of use of commonly consumed drugs, both legal (such as alcohol and tobacco) and illegal, as well as on treatment-seeking frequency, purpose of use and other related factors. Data from the EWSD have provided valuable insights for experts, researchers, and public health institutions to get valuable insights from these data (e.g., Gremeaux, 2022).

## Method

### Participants and Procedure

As stated before, this research was conducted within the broader context of the EWSD, a multinational, web-based targeted survey developed and coordinated by the European Union Drugs Agency and carried out by its national partners across 30 European and neighboring countries in 2024. The EWSD was created to serve as a more reliable tool for estimating the size of European drug markets by gathering data from people who use drugs about their consumption patterns and purchasing behaviors (Škařupová et al., 2019).

The survey was translated and adapted for local (Spain) use by the Spanish Observatory on Drugs and Addictions (Observatorio Español de las Drogas y las Adicciones). To take part, participants had to give their informed consent, be 18 years of age or older, and have used at least one of the following substances in the past year: cannabis, cocaine, ecstasy/MDMA, amphetamine, methamphetamine, heroin, or any new psychoactive substances.

We used the database from the EWSD, taking only data from Spanish respondents. The database used in this article corresponds to the 2024 survey. In this edition of the EWSD, data were collected between May and June 2024. The survey was advertised in various ways throughout the country, but most respondents found it through posts or paid advertisements on social media (European Union Drugs Agency, 2024). A total of 6399 valid respondents residing in Spain completed the survey anonymously.

### Measures

The EWSD included general modules that were presented to all participants, as well as specific modules for different substances, which were only shown to individuals who reported prior use of the respective substance (Matias, 2022). The EWSD questionnaire, organized into modules, consisted of 225 questions in total and took approximately 15 min to complete. The survey covered a range of topics, including sociodemographic data, drug use details (such as type, frequency, amount, setting, and method of use), treatment history, sources of supply, and prices.

### Statistical Analysis

We included all the sociodemographic variables to describe the sample. Regarding sex and gender, we took the information from two questions, asking for the assigned sex at birth and the gender identity. Those respondents who answered “male” to both questions were considered cis male, those who answered “female” in both questions were coded as cis female, and those who gave dissimilar answers in each question or answered “Transgender/non-binary” to the question about gender identity were grouped into the “transgender or non-binary” category. We performed descriptive statistical analysis of substance use, based on the questions regarding the context of use of each of the drugs or drug types included in the survey. We considered that a respondent was using a drug if they declared having consumed it in the last 12 months. Use of drugs for sex-related purposes was determined using these same questions, and then these groups were compared on the drug type used. Participants reported their substance use over the previous 12 months. For each reported substance, they specified the motives for use and the situations in which it occurred. “Chemsex party” was included as one of the predefined situational options. Individuals were categorized as engaging in chemsex if they indicated this context for at least one substance within the past year; all others were categorized as non-chemsex participants. We calculated percentages for qualitative variables and means and standard deviations for quantitative variables. We compared sociodemographic variables in SDU and non-SDU subgroups through t-tests (for quantitative variables) or chi-square tests (for qualitative variables). In the rest of the cases, when data from two or more subsamples were obtained, we obtained 95% confidence intervals. We used R version 4.3.2 software for all statistical analyses (R Core Team, 2023).

## Results

### Purpose and Contexts of Drug Use and Prevalence of Sex-Related Use

The survey was completed by a total of 6399 respondents residing in Spain who reported illicit drug use. Table 1 shows the distribution of the total sample according to the reported purposes of drug use. The most frequently used drugs for sexual purposes, including chemsex, were methamphetamines and cannabis.

**Table 1:** Reported purposes of drug use by substance

	Any drug	Cannabis	Cocaine (powder)	Cocaine (crack)	Ecstasy/MDMA	Amphetamines	Methamphetamines	Heroin	NPS	Ketamine
Relax or reduce stress	42.96%	49.73%	8.11%	0.72%	3.32%	2.47%	2.64%	30.14%	7.99%	10.98%
Improve sleep	26.66%	34.77%	NA	NA	NA	NA	NA	16.44%	4.96%	NA
Stay awake	13.05%	NA	13.36%	0.77%	7.25%	21.31%	8.15%	NA	5.63%	1.39%
Reduce the effect of alcohol	12.03%	NA	22.14%	0.60%	2.28%	9.57%	2.42%	NA	NA	0.58%
Manage depression / anxiety	26.35%	27.97%	8.35%	0.81%	2.99%	3.40%	2.20%	30.14%	5.55%	7.51%
Reduce pain	11.02%	12.59%	1.73%	0.49%	0.56%	0.80%	0.88%	17.81%	NA	3.70%
Manage health issues	0,44%	NA	NA	NA	NA	NA	NA	NA	2.35%	NA
Curiosity	21.82%	9.87%	9.38%	0.70%	17.14%	7.29%	6.39%	12.33%	18.08%	26.82%
Fun (Recreational)	78.01%	62.45%	61.54%	2.47%	73.71%	42.31%	31.05%	39.73%	25.90%	57.57%
Socialize	32.52%	22.57%	23.55%	0.63%	23.89%	14.64%	9.91%	9.59%	8.33%	10.64%
EnhancePerformance (school/work/sport/etc.)	8.85%	7.8%	2.93%	0.14%	0.47%	5.81%	1.76%	8.22%	2.52%	0.58%
Sex enhancement	13.66%	9.13%	5.96%	0.67%	8.14%	3.71%	10.35%	5.48%	5.21%	3.35%
Dependence	17.14%	22.36%	NA	NA	NA	NA	NA	27.40%	NA	NA
Spiritual reasons	4.53%	6.06%	NA	NA	NA	NA	NA	NA	NA	NA

This table shows the proportion of users of each specific drug based on the reasons they reported for its use. Reported purposes include recreational use, coping mechanisms (e.g., stress relief, managing anxiety or depression), social motives, sexual enhancement, among others. Percentages represent the proportion of users of each substance who indicated each respective motive. NA indicates cases where the sample size was insufficient or the response was not applicable

A total of 874 (13.66%) respondents reported having consumed drugs to enhance their sexual performance or experience at least once in the last 12 months. The number of male respondents who used drugs for sexual enhancement was approximately twice that of female respondents. Table 2 describes the context of use of each drug in this survey.

**Table 2:** Usage contexts of each drug (percentage over consumers of each drug)

	Cannabis	Cocaine (powder)	Cocaine (crack)	Ecstasy/MDMA	Amphetamines	Methamphetamines	Heroin	NPS	Ketamine
Music festival or party	49.81%	54.67%	0.76%	69.95%	42.31%	24.01%	9.59%	17.49%	47.28%
Club or bar	34.90%	51.89%	0.81%	41.18%	31.81%	13.88%	9.59%	12.20%	29.60%
Home	79.83%	49.00%	3.00%	21.31%	25.76%	17.84%	60.27%	25.82%	42.20%
Work	6.58%	7.47%	0.42%	0.50%	5.31%	2.20%	17.81%	2.10%	1.85%
School	7.76%	0.85%	0.11%	0.53%	1.36%	1.10%	4.11%	1.18%	1.16%
Car	16.47%	12.09%	1.20%	1.57%	5.00%	1.98%	20.55%	1.60%	3.93%
Chemsex party	1.51%	2.15%	0.35%	1.72%	1.73%	9.69%	2.74%	5.13%	3.93%
Drug consumption room	11.97%	1.62%	0.42%	0.65%	1.67%	2.20%	9.59%	0.93%	1.62%
Public space	54.65%	20.09%	0.99%	10.54%	11.74%	6.83%	21.92%	9.76%	12.72%
Nature	51.1%	5.96%	0.63%	5.86%	5.44%	4.41%	15.07%	7.91%	8.09%

### Sociodemographic Characteristics

Table 3 shows the sociodemographic characteristics. Statistical analyses revealed a significant sex difference assigned at birth and gender identity between sex-related drug users and other drug users (*p* = 0.004, and *p* < 0.001, respectively). Additionally, the percentage of transgender or non-binary individuals was slightly higher among sex-related drug users (3.66%) than in the rest of the sample (2.38%). Sex-related drug users were also younger than other drug users (*p* = 0.043). Employment status also showed a significant difference between the groups (*p* = 0.018).

**Table 3:** Sociodemographic characteristics of the total sample and subgroups of sex-related and non-sex-related drug users

	Absolute frequency and percentage/Mean and SD			Significance
	Whole sample (n = 6399)	SDU (n = 874)	Other drug users (n = 5525)	
**Sex assigned at birth**				*p* = 0.004
Male	4004 (62.65%)	594 (67.96%)	3408 (61.81%)	
Female	2307 (36.11%)	268 (30.66%)	2039 (36.98%)	
Intersex	5 (0.08%)	1 (0.11%)	4 (0.07%)	
Prefer not to answer	74 (1.16%)	11 (1.26%)	63 (1.14%)	
**Gender**				*p* < 0.001
Cis Men	3594 (61.90%)	575 (65.79%)	3370 (61.11%)	
Cis Women	2198 (34.41%)	248 (28.38%)	1950 (35.36%)	
Transgender or non-binary	160 (2.50%)	32 (3.66%)	131 (2.38%)	
Prefer not to answer	76 (1.19%)	3 (0.34%)	64 (1.16%)	
**Age**	31.42 (10.67)	30.75 (10.51)	31.52 (10.69)	*p* = 0.043
**Living situation**				*p* = 0.230
Couple				
Without children	1334 (22.13%)	181 (21.34%)	1153 (22.25%)	
With children	603(9.42%)	75 (8.58%)	528 (9.55%)	
Single				
Without children	1165 (19.32%)	174 (20.52%)	991 (19.13%)	
With children	191(2.98%)	22 (2.52%)	169 (3.06%)	
Living with parents	1724 (28.60%)	251 (29.60%)	1473 (28.43%)	
Sharing with peers	773 (12.82%)	103 (12.15%)	670 (12.93%)	
Without permanent residence	44 (0.73%)	12 (1.42%)	32 (0.62%)	
Other situation	195 (3.23%)	30 (3.54%)	165 (3.18%)	
**Level of Education**^**a**^				*p* = 0.750
Never went to school (ISCED 0)	18 (0.30%)	2 (0.24%)	16 (0.31%)	
Primary (ISCED 1)	285 (4.72%)	46 (5.41%)	239 (4.61%)	
Secondary (ISCED 2 to 3)	2978 (49.32%)	420 (49.35%)	2558 (49.32%)	
Higher Education (ISCED 4 to 8)	2757 (45.66%)	383 (45.01%)	2374 (45.77%)	
**Employment**				*p* = 0.224
Student	879 (14.56%)	135 (15.90%)	744 (14.34%)	
Working student	707 (11.71%)	123 (14.49%)	584 (11.26%)	
Employed	3375 (55.91%)	448 (52.77%)	2927 (56.22%)	
Employed part-time	1393 (23.07%)	206 (24.26%)	1187 (22.88%)	
Unemployed	357 (5.91%)	50 (5.89%)	307 (5.92%)	
Disability payment	54 (0.89%)	4 (0.47%)	50 (0.96%)	
Other	151 (2.50%)	18 (2.12%)	133 (2.56%)	
**Residency**				*p* = 0.114
City	2509 (41.57%)	388 (45.59%)	2121 (40.91%)	
Suburb	322 (5.33%)	39 (4.58%)	283 (5.46%)	
Town	1973 (32.69%)	268 (31.49%)	1705 (32.88%)	
Village/Countryside	1201 (19.90%)	152 (17.86%)	1049 (20.23%)	
Other	31 (0.51%)	4 (0.47%)	27 (0.52%)	
**Income**				*p* = 0.137
Minimum	1997 (33.42%)	309 (36.57%)	1688 (32.90%)	
Low	2604 (43.58%)	356 (42.13%)	2248 (43.82%)	
Low-medium	992 (16.60%)	121 (14.32%)	871 (16.98%)	
High-medium	238 (3.98%)	33 (3.91%)	205 (4.00%)	
High	75 (1.26%)	13 (1.54%)	62 (1.21%)	
Maximum	69 (1.15%)	13 (1.54%)	56 (1.09%)	

^a^ISCED: International Standard Classification of Education

Absolute frequencies and percentages/ Mean and SD. Significance values refer to the differences between sex-related drug users and other drug users (*p*-values from chi-square or *t*-test, as appropriate)

### Sexualized Drug Use by Drug Type and Gender Identity

Table 4 describes the percentage of users of each drug who used it for sexual enhancement. Cis men reported higher rates of sexualized use of cocaine (crack), heroin and MDMA compared to cis women. Additionally, transgender or non-binary individuals reported higher use of MDMA for sexual purposes than cis women, while cis men reported higher use of cocaine (crack) and heroin than transgender or non-binary respondents. Cannabis was the most frequently used substance for this purpose across all gender groups.

**Table 4:** Percentage of use of each drug for sexual enhancement

	Use to enhance sexual pleasure or performance (percentage over number of users of each drug)			
	Total sample	Cis Men (n = 705)	Cis Women (n = 93)	Transgender or non-binary (n = 60)
Cannabis	50.34% (47.02,53.66)	50.21% (46.52,53.9)	52.69% (42.54,62.84)	46.67% (34.05,59.29)
Cocaine (powder)	19.34% (16.72,21.96)	19.72% (16.78,22.66)	17.2% (9.53,24.87)	20% (9.88,30.12)
Cocaine (crack)	2.17% (1.2,3.14)	0.28% (0,0.67)	0% (0,3.96)	0% (0,6.02)
Ecstasy/MDMA	31.46% (28.38,34.54)	31.91% (28.47,35.35)	23.66% (15.02,32.3)	38.33% (26.03,50.63)
Amphetamines	6.86% (5.18,8.54)	7.23% (5.32,9.14)	6.45% (1.46,11.44)	5% (0,10.51)
Methamphetamines	5.38% (3.88,6.88)	5.11% (3.48,6.74)	6.45% (1.46,11.44)	6.67% (0.36,12.98)
Heroin	0.46% (0.01,0.91)	0.57% (0.01,1.13)	0% (0,3.96)	0% (0,6.02)
NPS	7.09% (5.39,8.79)	7.38% (5.45,9.31)	7.53% (2.17,12.89)	5% (0,10.51)
Ketamine	3.32% (2.13,4.51)	3.26% (1.95,4.57)	3.23% (0,6.82)	5% (0,10.51)

Percentages refer to the proportion of users of each specific drug who reported using it to enhance sexual pleasure or performance. Values in parentheses represent 95% confidence intervals. CM = Cis men; CF = Cis women; T = Transgender or non-binary

**Fig. 1:**
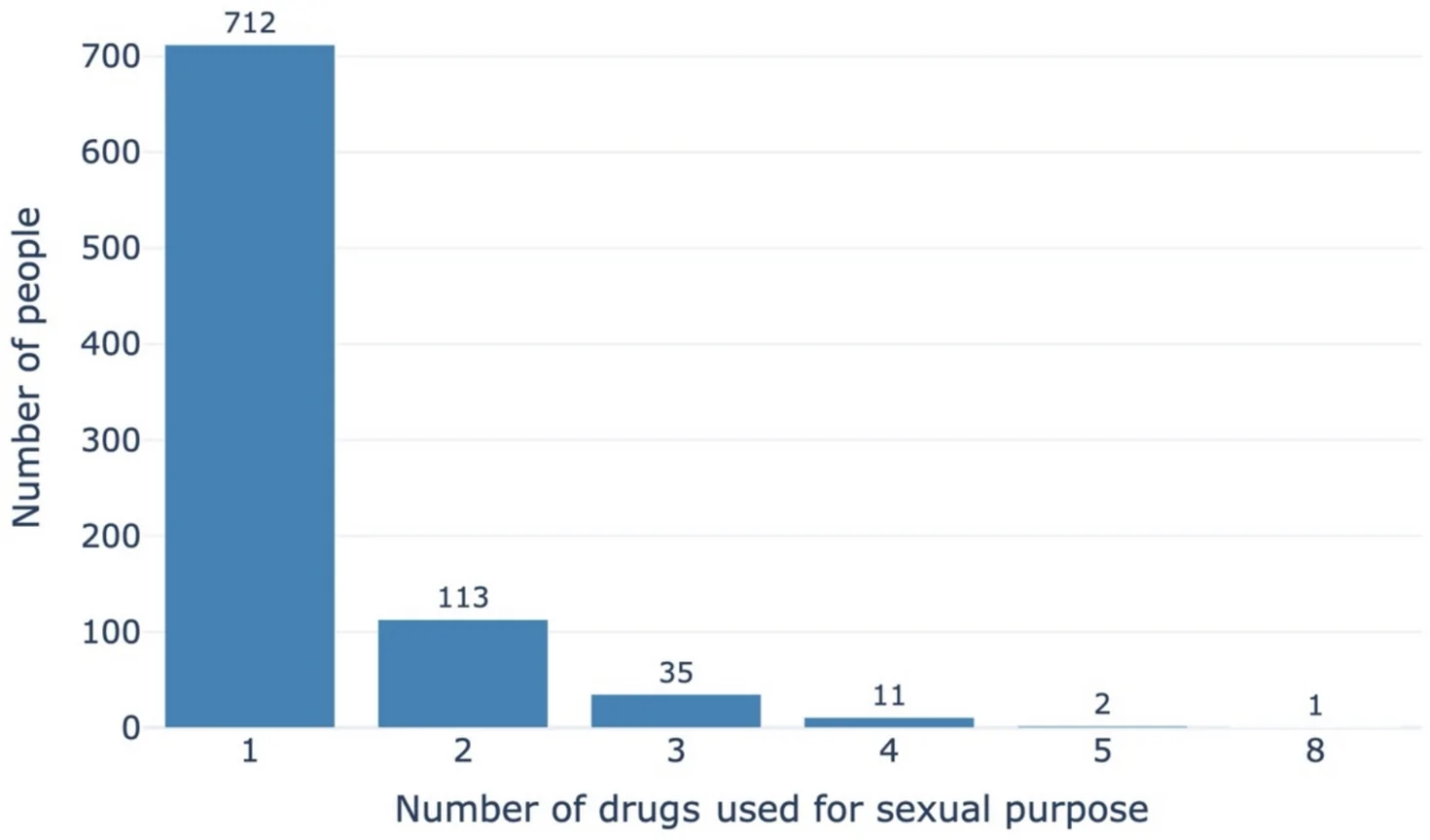
Number of people by number of drugs used for sexual purposes. Values represent the number of participants who reported using one or more different drugs to enhance sexual performance. The y-axis shows absolute frequency (n = 874)

Figure 1 illustrates the distribution of participants according to the number of different drugs used for sexual enhancement. Most respondents, 712 (81.46%), reported using only one substance. Furthermore, 113 (12.93%) participants consumed two or more substances (not necessarily at the same time, polydrug use), indicating that sexualized polydrug use is present within the sample, though the use of five or more substances was rare.

### Drug Use at Chemsex Parties by Gender

Table 5 describes the percentage of users of each drug used in chemsex. A total of 206 of the respondents attended at least one chemsex practice in the past 12 months. The majority were cis male (170; 81.33%), followed by cis female respondents (22; 10.53%), and transgender or non-binary individuals (14; 6.70%). Males reported higher use of CBD/Low-THC, heroin and ketamine compared to both females and transgender or non-binary individuals. However, cannabis use was higher in transgender or non-binary individuals than in cis female or male participants. Cocaine (crack) use was higher among cis females compared to both cis males and transgender or non-binary individuals, with cis males also reporting higher use than transgender or non-binary individuals. For ketamine, cis males report the highest use, followed by cis females and then transgender or non-binary individuals.

**Table 5:** Percentage of use of each drug in chemsex parties

	Total sample	Men (n = 170)	Women (n = 22)	Transgender or non-binary (n = 14)
CBD/Low-THC	10.48% (6.33,14.63)	12.84% (7.81,17.87)	5.56% (0,15.14)	0% (0,21.53)
Cannabis	34.76% (28.3,41.22)	35.13% (27.95,42.31)	27.78% (9.06,46.5)	66.67% (41.98,91.36)
Cocaine (powder)	29.05% (22.89,35.21)	28.37% (21.59,35.15)	31.48% (12.07,50.89)	33.33% (8.64,58.02)
Cocaine (crack)	4.76% (1.87,7.65)	3.38% (0.66,6.1)	9.26% (0,21.37)	0% (0,21.53)
Ecstasy/MDMA	27.62% (21.56,33.68)	27.7% (20.97,34.43)	25.93% (7.62,44.24)	33.33% (8.64,58.02)
Amphetamines	13.33% (8.72,17.94)	14.19% (8.94,19.44)	11.11% (0,24.24)	16.67% (0,36.19)
Methamphetamines	20.95% (15.43,26.47)	20.95% (14.83,27.07)	20.37% (3.54,37.2)	33.33% (8.64,58.02)
Heroin	0.95% (0,2.27)	1.35% (0,3.08)	0% (0,14.87)	0% (0,21.53)
NPS	29.04% (22.89,35.19)	29.05% (22.23,35.87)	31.48% (12.07,50.89)	16.67% (0,36.19)
Ketamine	16.19% (11.2,21.18)	12.84% (7.81,17.87)	5.56% (0,15.14)	0% (0,21.53)

Percentages refer to the proportion of users of each specific drug who reported using it in chemsex parties (percentage over the number of users of each drug). Values in parentheses represent 95% confidence intervals. CM = Cis men; CF = Cis women T = Transgender or non-binary

### Method of Drug Administration

Table 6 presents the distribution of drugs used and the methods of administration among chemsex practice attendees compared to the general population of drug users. Injection and rectal insertion were the most preferred administration methods among chemsex users compared to the general population. Smoking in pipes also appeared more prevalent among chemsex users for most drugs. In contrast, other routes such as snorting, vapor inhaling (“chasing the dragon”), and oral administration show less consistent patterns; for certain substances, these methods were more frequently reported by the general drug user than by chemsex users.

**Table 6:** Administration procedure of drugs by type among general users and chemsex party attendees

		Cocaine powder	Crack cocaine	MDMA	Amphetamine	Methamphetamines	Heroin	NPS	Ketamine
Snorting	All	77.26% (74.48,80.04)	0.82% (0.22,1.42)	14.15% (11.84,16.46)	45.83% (42.52,49.14)	13% (10.77,15.23)	13.7% (11.42,15.98)	16.49% (14.03,18.95)	65.55% (62.39,68.71)
	Chemsex	68.31% (64.88,71.74)	2.81% (1.59,4.03)	18.47% (15.61,21.33)	41.67% (38.03,45.31)	13.95% (11.39,16.51)	27.27% (23.98,30.56)	50.93% (47.24,54.62)	59.76% (56.14,63.38)
Injection	All	0.42% (0,0.85)	0.25% (0,0.58)	0.15% (0,0.41)	0.43% (0,0.86)	3.52% (2.3,4.74)	16.44% (13.98,18.9)	1.85% (0.96,2.74)	0.46% (0.01,0.91)
	Chemsex	2.11% (1.05,3.17)	2.12% (1.06,3.18)	1.91% (0.9,2.92)	3.7% (2.31,5.09)	13.95% (11.39,16.51)	27.27% (23.98,30.56)	15.74% (13.05,18.43)	1.22% (0.41,2.03)
“Chasing the dragon” (vapor inhaling)	All	0.6% (0.09,1.11)	0.88% (0.26,1.5)	0.18% (0,0.46)	0.06% (0,0.22)	1.98% (1.05,2.91)	45.21% (41.9,48.52)	0.76% (0.18,1.34)	0.23% (0,0.55)
	Chemsex	3.52% (2.16,4.88)	2.12% (1.06,3.18)	1.27% (0.44,2.1)	0.93% (0.22,1.64)	2.33% (1.22,3.44)	27.27% (23.98,30.56)	1.85% (0.86,2.84)	0% (0,0.68)
Oral dispensing	All	3.14% (1.98,4.3)	0.1% (0,0.31)	77.18% (74.39,79.97)	10.62% (8.57,12.67)	19.82% (17.17,22.47)	4.11% (2.79,5.43)	16.99% (14.5,19.48)	2.43% (1.41,3.45)
	Chemsex	5.63% (3.93,7.33)	1.41% (0.54,2.28)	64.33% (60.79,67.87)	12.04% (9.64,14.44)	6.98% (5.1,8.86)	9.09% (6.97,11.21)	15.74% (13.05,18.43)	2.44% (1.3,3.58)
Rectal insertion	All	0.25% (0,0.58)	0.04% (0,0.17)	0.15% (0,0.41)	0.25% (0,0.58)	1.32% (0.56,2.08)	1.37% (0.6,2.14)	1.01% (0.35,1.67)	0.35% (0,0.74)
	Chemsex	2.82% (1.6,4.04)	0.71% (0.09,1.33)	1.27% (0.44,2.1)	1.85% (0.86,2.84)	4.65% (3.1,6.2)	9.09% (6.97,11.21)	9.26% (7.12,11.4)	2.44% (1.3,3.58)
Smoked in pipe	All	NA% (NA,NA)	2.61% (1.55,3.67)	0.18% (0,0.46)	0.31% (0,0.68)	12.77% (10.55,14.99)	23.29% (20.48,26.1)	4.63% (3.23,6.03)	0.12% (0,0.35)
	Chemsex	NA% (NA,NA)	7.75% (5.78,9.72)	2.55% (1.39,3.71)	2.78% (1.57,3.99)	41.86% (38.22,45.5)	18.18% (15.33,21.03)	4.63% (3.08,6.18)	1.22% (0.41,2.03)

Percentages refer to the proportion of users of each specific drug who reported using that administration method. Values in parentheses represent 95% confidence intervals. “All” refers to the general population of drug users; “Chemsex party attendees” refers to participants who reported attending at least one chemsex party in the past 12 months. “Chasing the dragon” refers to vapour inhalation. NA = Not applicable or not reported due to insufficient data

## Discussion

The present study expands our understanding of SDU based on a large sample of respondents in Spain. The most extended use of all the drugs was for fun/recreational purposes by getting high. Frequencies of this recreational usage go from 73% of ecstasy/MDMA users to 25% of NPS users who did it to get high. For most of the drugs, socializing was the second most frequent use. Interestingly, the use pattern of cannabis and heroin was different from this general trend, as both substances were more commonly used for relaxation, stress reduction or improving sleep quality. In addition, both substances were widely used to manage symptoms of anxiety and depression. Furthermore, cannabis use was far more prevalent than heroin use. This finding was not unexpected, as cannabis is typically perceived as a relatively low-risk, low-harm substance (Harrison et al., 2024), a reputation that does not extend to heroin (Pedersen et al., 2017). Nonetheless, despite these contrasting risk perceptions, comparable proportions of users reported consuming both substances due to dependence.

Regarding the context of drug usage, all drugs were predominantly consumed at home, but there were public spaces commonly chosen for drug use. Among them, music festivals and parties were among the most frequent settings for drug use. Studies analyzing wastewaters have identified increased levels of drug use, mostly psychostimulants, during musical festivals, particularly those featuring rap, house, dance, pop, or rock music (Lai et al., 2013; Mackulak et al., 2019). Similarly, Lim et al. (2008) found that drug use was more prevalent among attendees of rap and house festivals. Consistent with our results, MDMA and cannabis were among the most used illicit drugs in music festivals (Benaglia et al., 2020; Hesse et al., 2010; Johnson et al., 2020). Slightly less frequent was the use of drugs in clubs or bars. Lastly, only cannabis and heroin were reported to be used across a wide range of environments, including schools, drug use rooms, and nature.

Across different uses and contexts, SDU emerges as a growing pattern of illicit drug use. The statistical analyses showing that SDU participants were disproportionately assigned male at birth, included a higher proportion of cisgender men, and were slightly younger and more often students are consistent with patterns reported in the SDU/chemsex literature. Several studies have shown that SDU and chemsex are concentrated among MSM and cisgender men, with substantially higher pooled prevalences in male sexual-minority samples than in general population groups (Georgiadis et al., 2025). Meta-analytic and epidemiological evidence also indicates elevated rates of substance use among transgender and gender-diverse individuals compared with their cisgender peers, which aligns with the modestly higher proportion of transgender and non-binary participants observed in the sex-related user group in our sample (Cotaina et al., 2022). Age-patterning in SDU frequently shows concentration among younger adults and sexual-minority youth, supporting our finding that sex-related drug users were, on average, slightly younger than other users (Ristuccia et al., 2018). Finally, reviews of chemsex and SDU emphasize considerable heterogeneity across studies for sociodemographic correlates beyond gender and age (for example, findings on housing, income, residence and education are inconsistent), which aligns with our null findings for living situation, income, residency and education in this sample (Mundy et al., 2025).

Findings that cisgender men reported higher sexualized use of stimulants, heroin, and MDMA than cisgender women are consistent with broader epidemiologic and clinical literature showing greater prevalence of stimulant and opioid use among men (McHugh et al., 2024). The observation that transgender and non-binary participants reported higher sexualized MDMA use relative to cisgender women, while cis men reported higher sexualized cocaine (crack) and heroin use than transgender/non-binary respondents, likewise accords with evidence that substance use patterns differ across gender-diverse groups, with some substances (including stimulants and party drugs such as MDMA/ecstasy) showing elevated prevalence in transgender and gender-diverse populations compared with cisgender peers (Cotaina et al., 2022). Finally, the prominence of cannabis as the most frequently used substance for sexual purposes across all gender groups mirrors population-level and review data identifying cannabis as the most used illicit drug overall in studies of recreational and contextual (including sexual) use (Calakos et al., 2017).

The distribution showing that most participants reported using only one drug for sexual enhancement, with a smaller proportion engaging in multi-substance use, aligns with previous research on SDU and chemsex behaviors. For example, a recent systematic review and meta-analysis found that among MSM, the pooled prevalence of SDU was 25%, with specific substances such as cocaine (10%) and ecstasy/MDMA (9%) (Georgiadis et al., 2025). This work underscores that single-substance use remains the dominant pattern. Another study of sexually transmitted infections‐clinic attendees in Spain reported that 33.8% of respondents reported SDU (ie, “chemsex” defined broadly), and among them, the most frequently reported drugs were poppers and cannabis (20%), with cocaine, mephedrone and ecstasy also present (Rosas Cancio-Suarez et al., 2023). This aligns with our observation that cannabis or “lighter” substances may dominate, and that extensive polydrug use (five or more) is rare. In any case, our finding of 13% of participants using two or more drugs indicates a meaningful subgroup with polydrug sexualized use, a subgroup which prior work suggests may have elevated risk (i.e. sexually transmitted infections, adverse sexual or mental health outcomes) compared to single-substance users (Gonzalez-Baeza et al., 2018).

Regarding our findings about the percentage of users of each drug consumed in chemsex, broken down by sex assigned at birth and gender identity, these patterns align with extant literature indicating that chemsex and SDU contexts are marked by gendered differences in substance choice. For instance, one large UK cross‐sectional study among MSM found that stimulants and sex-enhancement drugs were disproportionately used among cisgender men relative to other groups (Sarwar et al., 2023). Furthermore, gender‐diverse populations (including transgender and non-binary individuals) are under-represented in chemsex research, but available qualitative reviews indicate they may engage with different drugs or contexts (for example, cannabis as a more accessible substance) and have specific social/structural drivers of use (Kelly-Hanku & Newland, 2021). The higher cannabis use among transgender/non-binary individuals in our study, compared to cisgender groups, may reflect these factors: accessibility, cost, lower stigma or risk perceptions, or therapeutic/affective motivations, rather than purely enhancement-driven use, among others. Meanwhile, the elevated heroin, ketamine and low‐THC product use among cis males may reflect sub-cultural dynamics of chemsex linked to longer sessions, group sex, or drug‐sex networks that are more prevalent in cis male populations.

Our findings indicating distinct route-of-administration profiles (greater injection and rectal insertion, greater smoking/pipe use) among chemsex attendees compared to general drug-users are empirically grounded. Intentional injection in chemsex contexts has been documented as a higher‐risk variant of SDU. For example, a Spanish national technical document reports that among those who had injected in the previous year, a large proportion used stimulants associated with chemsex (Methamphetamine, Mephedrone) and links injection specifically with chemsex practices (Ministerio de Sanidad, 2020). Furthermore, a study of MSM individuals found that a greater number of routes of administration predicted a higher frequency of chemsex practice (Sanchez-Ocaña et al., 2024). Our observation that smoking/in-pipe use is relatively more prevalent among chemsex users also resonates with qualitative accounts of “party & play” sessions, where smoked or inhaled routes (e.g., for methamphetamine, mephedrone) may facilitate rapid onset, prolonged sexual sessions and group dynamics. Conversely, the fact that other routes such as snorting, vapor inhalation (chasing the dragon), and oral administration show less consistent patterns, and in some substances are more common among general users than among chemsex‐users, is also coherent. The systematic review among MSM reported above indicates substantial heterogeneity in substances and does not specifically report much on route differences, but underscores that administration routes matter and differ by context (Georgiadis et al., 2025).

Finally, drug use in chemsex is still minoritarian among drug users, except for methamphetamine and NPS. Methamphetamines were mostly consumed by males to enhance sexual performance or pleasure. The pattern of use reported here, with methamphetamine and NPS predominantly used in chemsex, is consistent with prior research (Dolengevich-Segal et al., 2017; González-Recio et al., 2024). Although chemsex is typically associated with men and MSM, a minority of women from our sample also took part in these activities. This finding suggests that the chemsex practices may be changing. Although empirical studies on heterosexual men and women are very limited, there is a growing interest in studying these groups (Lawn et al., 2019; Miltz et al., 2021). Further research on this area is warranted. This will help researchers to update information and develop intervention strategies, as already proposed (Pirani et al., 2019).

### Strengths and Limitations

The major strength of this study is the large sample of participants, which provides sufficient statistical power for all analyses. However, several limitations should be acknowledged. First, the cross-sectional design prevents us drawing firm conclusions about causal relationships or the evolution of drug use patterns over time. While we attempted to address this by examining patterns across age groups, such interpretations should be made with caution. Second, the survey did not explore in detail the specific types of novel psychoactive substances (NPS, poppers, etc.) being consumed. Future research should aim to clarify which NPS are being used. Finally, all data were based on self-reported measures, without biological verification of drug use, which may introduce bias due to underreporting or misreporting.

### Conclusion

Illicit drug use occurs predominantly for recreational purposes. Consumption most frequently takes place in private settings such as the home, but also in public spaces like music festivals, clubs, and bars. Although chemsex has traditionally been associated with MSM, it is increasingly emerging within heterosexual populations. This shift in the epidemiology of chemsex underscores the need to adapt prevention and risk-reduction campaigns, which have until now focused primarily on MSM communities. This practice entails substantial physical, sexual, and mental health risks. Addressing these challenges requires comprehensive intervention and prevention strategies, including public education, accessible healthcare services, community-based support, harm-reduction initiatives, and specialized training for professionals. These measures are essential to reduce the harms associated with chemsex and improve overall health outcomes.

## Data Availability

The data used in this study were obtained from the European Web Survey on Drugs, a governmental source managed by the European Monitoring Centre for Drugs and Drug Addiction (EMCDDA). The dataset is not publicly available, as it is not an open database, and access is restricted to authorized institutions. Therefore, the authors are not permitted to share the raw data. Interested parties may request access directly from the EMCDDA.

## Code Availability

We used R version 4.3.2 software for all statistical analyses (R Core Team, 2023).
